# Characteristics of Carbapenem-Resistant Gram-Negative Bacilli in Patients with Ventilator-Associated Pneumonia

**DOI:** 10.3390/antibiotics10111325

**Published:** 2021-10-29

**Authors:** Amira Mohamed, Enas Daef, Amany Nafie, Lamia Shaban, Maggie Ibrahim

**Affiliations:** 1 Clinical Pharmacy, Women’s Health Hospital, Assiut University, Assiut 71711, Egypt; amirayousif10@gmail.com; 2 Microbiology and Immunology Department, Faculty of Medicine, Assiut University, Assiut 71711, Egypt; deafenas@yahoo.com (E.D.); amanynafeh@yahoo.com (A.N.); 3 Chest Department, Faculty of Medicine, Assiut University, Assiut 71711, Egypt; lamiashaban@yahoo.com

**Keywords:** carbapenem-resistance, ventilator-associated pneumonia

## Abstract

Carbapenem-resistant Gram-negative bacilli (CR-GNB) has become a global threat. In hospital settings, the association of CR-GNB with ventilator-associated pneumonia (VAP) is a critical public health concern owing to their high resistance rate to most antibiotics. The present study aims to identify the frequency of carbapenem-resistance and to determine the rate of multidrug resistance (MDR), extensive drug resistance (XDR) and pan-drug resistance (PDR) among CR-GNB infections in VAP. Antimicrobial susceptibility testing was carried out using the disk diffusion method and the detection of carbapenemases was screened using the imipenem-E test and the modified carbapenem-inactivation method (mCIM). The isolates were verified by polymerase chain reaction (PCR) for the presence of *bla*_NDM_, *bla*_SPM_, *bla*_VIM_, *bla*_IMP_ and *bla*_GIM_ genes. 89.5%, 14%, 17.5%, 10.5%, 3.5% of isolates exhibited the presence of *bla*_NDM_, *bla*_VIM_, *bla*_SPM_, *bla*_IMP_ and *bla*_GIM_, respectively. 76%, 17% and 7% of isolates were PDR, XDR, and MDR, respectively. Carbapenem-resistance genes were identified in a significant percentage and *bla*_NDM_ was the most predominant gene. All isolates were highly resistant to most antibiotics. This health concern has proven to be a big challenge in developing countries such as Egypt, as it is associated with high morbidity, high mortality, and raised healthcare costs.

## 1. Introduction

Most seriously sick patients admitted to intensive care units (ICUs) reveal bad nutritional and general conditions, down immune status, prolonged hospital stays, and a history of invasive procedure and, consequently, they are vulnerable to increased propensity to infection [1].

Regardless of the various complications related to mechanical ventilation (MV), it is lifesaving for most of ICUs patients. One of these complications is ventilator-associated pneumonia (VAP) which is a highly prevalent hospital-acquired infection (HAI) in ICUs. VAP is allied with lengthy hospital and ICU stays, prolonged MV time, expanded hospital expenses, enduring morbidity, and increasing mortality [2].

Antimicrobial resistance (AMR) is a worldwide health concern due to its correlation with high morbidity and mortality and raised healthcare costs [3]. The problem of AMR is increasing globally with the spread frequently striking healthcare settings due to the improper use of antimicrobials and inappropriate infection control practices. AMR risk is especially in low- and middle-income countries [4].

Carbapenem-resistant Gram-negative bacilli (CR-GNB) are a threat worldwide. Carbapenem-resistant Enterobacterales (CRE), carbapenem-resistant *Pseudomonas aeruginosa* (CRPA), and carbapenem-resistant *Acinetobacter baumannii* (CRA) are considered as carbapenem-resistant organisms (CROs) [5]. Enterobacterales is an order of Gram-negative bacteria, comprising seven families and more than 80 genera. It incorporates pathogens accountable for 30% of HAIs [6]. The global priority list of pathogens by the World Health Organization (WHO) has categorized CRE, CRPA, and CRA as critical or highest priority [7].

Carbapenems have been the drugs of choice to cure critical VAP infections, although illogical and very common use of carbapenems has given rise to the emergence of CR-GNB strains. Resistance to carbapenems is mediated by a variety of mechanisms [4]. Carbapenemase enzymes, the most significant of these mechanisms, are β-lactamases that inactivate many β-lactam antibiotics. Carbapenemases belong to three classes amongst the four classes of β-lactamases defined by the Ambler classification. Class A contains serine in the active site of the enzyme (e.g., SME, IMI, NMC, GES and KPC), class B, metallo- beta- lactamases (MBLs) (e.g., IMP, NDM, VIM and GIM); and class D (OXA-like). Lately, it has become challenging to treat VAP because of the emergence of class B MBLs, which possess potent carbapenemase activity and are wide-ranging and resistance to almost all β-lactam antibiotics [8]. Gene coding for many carbapenemases are located on mobile genetic elements (MGEs), which could be spread between strains. Moreover, these MGEs often carry further genes that provide resistance to non-β-lactam antibiotics, further limiting treatment options for CR-GNB infections. It is therefore logical that carbapenem use would lead to the emergence of resistance [6,9,10,11].

Multidrug resistant bacteria (MDR) present a certain risk in hospitals, especially among patients who need procedures such as blood catheters and ventilators as they are more susceptible to serious and frequently fatal infections, such as bloodstream infections and pneumonia. These bacteria have developed a resistance to many antibiotics, including carbapenems and third generation cephalosporins which are the greatest available antibiotics for MDR bacteria. MDR implies resistance by the strains to three or more antimicrobial drug classes. Extensive drug resistance (XDR) implies resistance to all but two drug classes. Pan-drug resistance (PDR) implies resistance to all drug classes [12]. VAP has high risk of mortality and morbidity and may be attributable to inappropriate or delayed empirical antibiotic therapy, particularly when VAP is produced by MDR [13].

Our study was conducted to identify the frequency of CR-GNB infections in VAP patients at a chest and women’s health hospital (WHH) ICUs, Assiut University Hospitals, Egypt, and to determine the frequency of MDR, XDR, and PDR among CR-GNB causing VAP.

## 2. Results

### 2.1. Identification of the Bacterial Isolates

Seventy-five pathogens were isolated from VAP patients. Of these, 57 (76%) were Gram-negative bacilli, 16 (21.4%) were Gram-positive cocci, and 2 (2.6%) were *Candida* spp. Out of 57 Gram-negative bacilli isolates, *Klebsiella pneumoniae* was the most common isolate (*n* = 32), *Klebsiella oxytoca* (*n* = 1), *E. coli* (*n* = 5), *Proteus* *vulgaris* (*n* = 4), *Pseudomonas aeruginosa* (*n* = 11), and *Acinetobacter* *baumannii* (*n* = 4).

### 2.2. Antimicrobial Susceptibility Testing

The isolates showed 100% resistance to both ampicillin and cefaclor. They were highly resistant to meropenem (94.7%), ciprofloxacin and norfloxacin (92.9% for each), ceftriaxone (94.7%), tobramycin (89.4%), and imipenem (80.7%). The anti-microbial susceptibility pattern of the isolates using the disk diffusion method is demonstrated in Table 1 and Table 2.

### 2.3. Phenotypic Carbapenemase Detection

E-test results showed that 36/57 (63.1%) isolates were resistant to imipenem with MIC value ranging from 0.002–32 mg/L.

The results of mCIM (using both meropenem and imipenem discs) revealed high level of resistance indicating potential carbapenemase production. Out of 57 isolates there were 43 (75.4%) imipenem-resistant isolates and 36 (63.1%) meropenem-resistant isolates.

The degree of agreement between disc diffusion method, E-test and mCIM for imipenem is shown in Table 3. There was a moderate significant agreement in the results as kappa level for E-test = 0.498 and for mCIM = 0.439 and *p*-value < 0.001.

### 2.4. Detection of Carbapenem Resistance Genes

The isolates were tested by PCR for detection of *bla*_NDM_, *bla*_VIM_, *bla*_SPM_, *bla*_IMP_, and *bla*_GIM_ genes. The frequency of the evaluated genes was: *bla*_NDM_ (89.5%), *bla*_VIM_ (14%), *bla*_SPM_ (17.5%), *bla*_IMP_ (10.5%) and *bla*_GIM_ (3.5%). *bla*_NDM_ was the most predominant gene. The co-existence of carbapenemase-producing genes (*bla*_NDM,VIM,SPM,GIM_) was detected in 19 strains (14 strains had 2 genes, 4 strains had 3 genes, and 1 strain had 4 resistance genes). 33 strains had only one resistance gene (32 strains had *bla*_NDM_ and one strain had *bla*_IMP_). While 5 strains did not have any resistance gene. Table 4 demonstrates the genetic profile of the isolated strains.

The association between carbapenem-resistant genes and imipenem-resistant strains (regarding mCIM) is shown in Table 5 and the association between carbapenem-resistant genes and meropenem-resistant strains (regarding mCIM) is shown in Table 6.

The degree of agreement between genotypic and phenotypic methods for detection of resistant strains is demonstrated in Table 7. There was a slight significant agreement between PCR and E-test as kappa level = 0.193 and moderate significant agreement between PCR and disc diffusion test.

Analysis of the basic medical history, history of antibiotic use, immune status, prolonged intubation, reintubation, invasive operation, and length of hospital stay was performed. The results revealed that the incidence of carbapenem-resistant *K. pneumoniae* (CRKP) was slightly higher in patients aged more than 50 years and increased with a longer hospital stay.

## 3. Discussion

The present study aims to identify the frequency of CR-GNB infections in VAP patients and to determine the frequency of MDR, XDR and PDR among CR-GNB causing VAP infections, especially since the rapid and precise detection of carbapenemase-producing isolates has proven difficult for laboratories and entails phenotypic and genotypic analysis.

In this study, 57 Gram-negative strains were isolated from VAP patients and amongst them *Klebsiella* spp. was the predominant isolate (*n* = 33, 57.9%). This correlates with a previous study in Egypt which declared that the most common pathogen for CR-GNB cases was *Klebsiella* spp. followed by *E. coli* [14]. However, our results were slightly higher than the incidence observed by Mohamed et al. Their samples were collected from patients admitted to different wards at Assiut University Hospital in Egypt. They declared that *Klebsiella* spp. was among the most common pathogens in intensive care units with a prevalence of (*n* = 16, 48.48%). Our different findings may be due to the fact that our samples were taken only from VAP patients, and that *K. pneumoniae* has been revealed as the major organism causing hospital-acquired infections involving pneumonia [15].

In agreement with our results, Gajdacs et al. detected 50 CR-GNB isolates; the majority of which were *Klebsiella* spp. (*K. pneumoniae* (*n* = 34), *Klebsiella oxytoca* (*n* = 1)), while *E. coli* (*n* = 12) and *E. cloacae* (*n* = 3) [16]. On the other hand, Rezai et al. in Iran found that it was not *K. pneumoniae* but *Pseudomonas aeruginosa* with an incidence of 24.4% with the most significant pathogen causing VAP; this could be attributed to the geographical difference between the countries [17].

In the current study the prevalence of *K. pneumoniae* was slightly higher in patients aged more than 50 years, but this was not statistically significant compared to those less than 50 years; 53.8%, 57.1% and, 62.5% of VAP patients stayed in the hospital ≤7 days, (8–14) days, >14 days, respectively, and the prevalence of *K. pneumoniae* increased with longer hospital stay, but different durations of hospital stay were statistically insignificant. Additionally, Kotb et al. in Egypt stated that a longer ICU stay prior to specimen collection was significantly associated with carbapenem resistance [14]. However, our findings were higher than the incidence observed by Khairy et al. in Minia, Egypt. Their study showed that (37.5%) of patients were reported with a length of hospital stay (LOS) >10 days, followed by (28.2%) with 8–10 days [18]. This may be attributable to the spread of resistant strains of *K. pneumonia* among VAP patients and the improper use of combined antibiotics as empirical treatment.

In the present study, 100% of isolates were resistant to both Cefaclor and Ampicillin. This agreed with a recent study conducted in Ethiopia. They found that 95 (35.2%) out of 270 patients in the ICU have got infections with ESBL and carbapenemase releasing Gram-negative bacilli (GNB) and all GNB isolates were 100% resistant to both tetracycline and ampicillin [19]. We also found that 94.7% of isolates were resistant to ceftriaxone, even though, in a previous study in Iraq in 2016 the rate of resistance was 95.83% for ampicillin and 79.17% for ceftriaxone [20]. A study in Iran revealed that 15.53% of the *P. aeruginosa* and 97.4% of the *A. baumannii* isolates were screened as imipenem-resistant [21]. Another recent study in Zambia detected imipenem-resistance in 6% of *P. aeruginosa* and 18.2% of *Acinetobacter* species isolates [22]. In contrast, we found that 90.9% of the *P. aeruginosa* and 50% of *A. baumannii* isolates were imipenem resistant. Our findings almost concur with a study in Korea, which stated that the antibiotic resistance profile of CRKP was 29/45 (64.4%) for imipenem and 36/45 (80.0%) for meropenem [23]. Due to the rapid and different protocols for antibiotics use, there is a variation between our findings and the described studies.

The current study revealed that 76% of isolates were pan-drug resistant, 17% were extreme-drug resistant, and 7% were multidrug resistant. In addition, another study in Egypt detected that all 50 CRKP isolates were multidrug resistant (MDR) [24]. These values may be attributable to the absence of an appropriate antibiotic strategy used for VAP patients, ensuing long and improper use of antimicrobial agents, which has ultimately resulted in marked antibiotic resistance. Management based on empirical broad-spectrum antibiotics and the widespread use of over-the-counter antibiotics in Egypt have led to massive selection pressure and an MDR crisis that is likely to become considerably worse in the perspective future.

E-test was performed for imipenem, and we observed that 36.9% (21/57 isolates) were imipenem-resistant. By performing mCIM test we also detected that 24.6% (14/57 isolates) and 36.9% (21/57 isolates) were resistant to both imipenem and meropenem, respectively. In the USA, Pierce et al., found that 91 of the 92 isolates characterized as bearing carbapenemase genes showed positive results by mCIM [25]. This difference can be explained by the fact that all the isolates in the USA study were formerly characterized as bearing carbapenemase genes. Another possible explanation is that mCIM might have missed few carbapenemase producers, which probably possessed low-level of carbapenemase activities.

Although disc diffusion is a preliminary method with lower sensitivity than other methods, we observed a moderate significant agreement between it and both E-test and mCIM for imipenem (kappa level for E-test = 0.498 and for mCIM = 0.439 and *p*-value < 0.001). The mCIM test is simple, low cost, does not require any equipment, and is easy to interpret. However, the relative long incubation time (from 8 hr to overnight) cannot be ignored. Another limitation for mCIM is that it is incapable of verifying the carbapenemase class.

We noticed a discrepancy between *bla*_NDM_-producing isolate and mCIM, which produced an indeterminate result (counted as negative), in contrast to a previous study in New Zealand, they found the discrepancy between *bla*_VIM_-producing isolate and the mCIM [26].

The genotypic analysis revealed that, 91.2% (52/57) of isolates had at least one resistance gene. However, it is very likely that the existence of only one carbapenem-resistance gene is not necessary associated with carbapenem-resistance. Contrary to what was previously noted in a study performed in Sudan, which revealed that 83% of isolates were positive by phenotypic tests, while 58.7% had one or more carbapenem-resistance genes [27].

In this study 89.5%, 14%, 17.5%, 10.5%, 3.5% of 57 isolates exhibited the presence of *bla*_NDM_, *bla*_VIM_, *bla*_SPM_, *bla*_IMP_ and *bla*_GIM_, respectively, and *bla*_NDM_ was the predominant gene by 89.5% (51/57 isolates). Our findings are consistent with previous studies in Egypt. Tawfick et al. detected that *bla*_NDM_ was the most prevalent genotype of carbapenemases by 68.88% (93/135). Furthermore, El-Domany et al. stated that the incidence of *bla*_NDM_ was 70.0% [24,28]. On the other hand, Ghaith et al., detected that the most prevalent carbapenemase gene was *bla*_OXA-48_ which was identified in 14/23 (60.8%) isolates followed by *bla*_NDM_, which was identified in 12/23 (52.2%) isolates [29]. Additionally, in a previous study in Assiut, El-rehewy et al. stated that 17.92% out of 97 isolates were positive to *bla*_IMP_ gene and 16.42% were positive to *bla*_VIM_ gene [30], even though our results differ from a published study in Sudan, which stated that *bla*_IMP_ gene was harbored by 6.7% of carbapenem-resistant isolates [31]. In India, Kumari et al. stated that 58.6% of VAP patients had high resistance to carbapenems. Of their 172 isolates, 21.5% exhibited the presence of *bla*_NDM_ genes, 17.4% exhibited the presence of *bla*_VIM_ gene and 8.7% isolates harbored both *bla*_NDM_ and *bla*_VIM_ genes. None of the isolates contained *bla*_IMP_ gene [32]. Sękowska et al. confirmed that *bla*_NDM_ and *bla*_VIM_ genes are the main antibiotic-resistance genes that induce resistance patterns to carbapenems among VAP patients in Poland [9]. Another study in Thailand reported that *bla*_NDM_ was the most common carbapenen-resistant gene, present in 65% isolates [3]. These concur well with our findings. However, Yan et al. in China detected that 89% of the carbapenen-resistant isolates were carbapenemase gene- positive, including 70% *bla*_KPC_, 13% *bla*_NDM_, 6% *bla*_IMP_, and 1% combined *bla*_KPC_/*bla*_NDM_ genes [4].

CRKP plays a major role in VAP. In this study, *bla*_NDM_ was the most prevalent gene among *K. pneumoniae* isolates. Concurring with our finding, a study in India found *bla*_NDM_ in 28.8% and *bla*_VIM_ in 17.7% of *K. pneumoniae* isolates among VAP patients [32]. However, a study from Iran revealed that *bla*_VIM_ was the most prevalent gene (13.6%), while both *bla*_IMP_ and *bla*_NDM_ were only detected in two *K. pneumoniae* isolates [33], which is not in concordance with our findings.

All *E. coli* isolates in the present study exhibited the presence of *bla*_NDM_ gene. This is in accordance with another study in China which stated that the most prevalent carbapenemase gene among *E. coli* isolates was *bla*_NDM_ [34].

In the current study, *P. aeruginosa* isolates harbored *bla*_NDM_ (90.9%) followed by *bla*_VIM_ (54.5%) and *bla*_SPM_ (18.2%). Similarly, in a study in Iran, *bla*_NDM_ was the most prevalent gene among *P. aeruginosa* isolates [35]. Additionally, high-levels of carbapenem-resistance induced by *bla*_NDM_ and *bla*_VIM_ were found in *A. baumannii* and *P. aeruginosa* isolates in a Libyan hospital [36]. A study in Saudi-Arabia detected *bla*_VIM_ and *bla*_GIM_ alone in 46% and 15% of *P. aeruginosa* isolates and *bla*_NDM_ was not detected in any of the isolates. These results contradict our findings regarding *bla*_NDM_ and *bla*_GIM_ [37].

In the present study, *Acinetobacter* isolates harbored *bla*_NDM_ (75%) and *bla*_IMP_ (50%). Another study corroborates with our results, as *bla*_NDM_ was detected in 65.6% of *Acinetobacter* isolates among VAP patients [38].

Despite being a global issue, there is a disparate distribution of AMR among countries, with more impact on developing countries [13,15,17]. This difficult burden on developing countries may be attributed to inadequate approaches to new antibiotics, expanded economic burden, and inability to afford second-line antibiotics, which may be more costly, thus producing worse outcomes. Moreover, in developing countries, improper infection prevention and control (IPC) measures have increased the incidence of AMR [39].

Limitations to the current study include the relatively small sample size and the lack of multiple gene types. Unfortunately, we were unable to perform whole-genome sequencing because of resource inaccessibility.

## 4. Materials and Methods

### 4.1. Specimen Collection

Study samples were collected over a period of 9 months (from May 2019 to January 2020) from chest and WHH ICUs, Assiut University Hospitals. Only patients showing bacteriologically verified pneumonia were investigated; establishing etiologic diagnosis required the isolation of bacteria in considerable quantity from lower respiratory tract secretions samples.

The study was performed on 35 VAP patients, 23 (66%) of them were males and 12 (34%) were females. The median age of studied patients was (60 (20–85) years old) with a mean age of 60.4 ± 15.7. The median of the length of ICU stay was 12 (5–30) days. All patients received antibiotics before the collection of samples; 9, 17, and 9 patients received antibiotics for ≤7 days, (8–14) days, and >14 days, respectively, and 10 (28.6%) patients received combined antibiotics during their hospital stay.

### 4.2. Identification of the Recovered Bacterial Isolates

Isolates were categorized based on their Gram reactions. Gram-negative isolates were selected for further study. Culture characteristics on Nutrient agar, Blood agar, Mac-Conkey’s agar, and Eosin Methylene Blue (EMB) agar were recorded. Biochemical tests, including urease test, oxidase test, Triple sugar iron agar and citrate utilization test were performed. Identification was confirmed using API20E kits (Liofilchem, Roseto, Italy).

### 4.3. Antimicrobial Susceptibility Testing

Antimicrobial susceptibility testing was conducted using the disc diffusion method according to CLSI instructions [40]. The following antimicrobial discs (Oxoid, Basingstoke, Hampshire, UK) were used: Ampicillin (10 μg), Cefaclor (30 μg), Ceftriaxone (30 μg), Tobramycin (10 μg), Ciprofloxacin (5 μg), Norofloxacin (10 μg), Imipenem (10 μg), Meropenem (10 μg).

### 4.4. Phenotypic Carbapenemase Detection

All isolates were tested for accurate determination of their MIC using the imipenem-E test strips (Liofilchem, Roseto, Italy). Results were interpreted using CLSI breakpoints. A cut-off point of ≥4 μg/mL was to define imipenem-resistance and a cut-off point of ≤1 μg/mL was to define imipenem-susceptibility [40].

The modified carbapenem-inactivation method (mCIM) was used by both meropenem and imipenem discs. 1 µL calibrated loopful of organism was suspended in 2 mL trypticase soy broth (TSB) and the suspension was vortexed for 15 s. Then, 10-μg meropenem or imipenem disc (Oxoid, Hampshire, UK) was added into the suspension aseptically. The suspension including the disc was subsequently incubated at 35–37 °C for 4 h. Then meropenem or imipenem disc was eliminated from the suspension using a 10-μL inoculating loop; the loop was dragged along the edge of the tube to remove excess liquid during removal, and instantly put on Mueller-Hinton (MHA) plate which was inoculated with a 0.5 McFarland suspension of standard carbapenem-susceptible *E. coli* strain ATCC^®^ 25,922 (mCIM indicator organism). The plates were incubated at 35–37 °C overnight; the inhibition zone around the disc was measured. Inhibition zone 6–15 mm or existence of colonies in a 16–18 mm zone was considered carbapenemase positive. While inhibition zone ≥ 19 mm was considered carbapenemase negative [25].

### 4.5. Molecular Testing for Carbapenemase Genes

Amplification of carbapenem-resistance encoding genes (*bla*_NDM,VIM,SPM,IMP,GIM_) was carried out by PCR using the proper primers (Table 8) [30,41,42,43]. Primers were manufactured by (Thermo Fisher Scientific, Waltham, MA, USA). The amplicons were analyzed by agarose gel electrophoresis, and the expected DNA product size was determined by comparing with an 1100 bp DNA ladder (Willowfort, Birmingham, UK).

## 5. Conclusions

This work has highlighted that CR-GNB, especially CRKP, caused a considerable percentage of VAP infections in chest and WHH ICUs, Assiut University hospitals. Our data revealed high levels of resistance to most antibiotics. Carbapenem-resistance genes were identified in a significant percentage of isolates and *bla*_NDM_ was the most predominant one.

This serious issue emphasizes the urgent need to implement evidence-based IPC strategies to prevent CR-GNB transmission and incorporate antibiotic stewardship programs in order to decrease the CR-GNB burden. In an era of increasing antimicrobial bacterial resistance, rapid identification of carbapenemase-positive strains is not only critical in preventing spread, but also an epidemiological and economic issue, especially in developing countries such as Egypt.

## Figures and Tables

**Table 1:** Antibiotic resistance profile of isolates from chest ICU.

Antimicrobial Agent	*K. pneumoniae* *n* = 29	*K. oxytoca* *n* = 1	*E. coli* *n* = 3	*P. vulgaris* *n* = 3	*P. aeruginosa* *n* = 10	*A. baumannii* *n* = 4
Ampicillin	100%	100%	100%	100%	100%	100%
Cefaclor	100%	100%	100%	100%	100%	100%
Ceftriaxone	100%	100%	66.7%	66.7%	100%	100%
Imipenem	89.6%	100%	100%	66.7%	100%	50%
Meropenem	96.5%	100%	100%	100%	100%	100%
Ciprofloxcin	100%	100%	100%	100%	100%	100%
Norfloxacin	100%	100%	100%	100%	100%	100%
Tobramycins	100%	100%	100%	100%	100%	100%

**Table 2:** Antibiotic resistance profile of isolates from WHH ICU.

Antimicrobial Agent	*K. pneumoniae* *n* = 3	*E. coli* *n* = 2	*P. vulgaris* *n* = 1	*P. aeruginosa* *n* = 1
Ampicillin	100%	100%	100%	100%
Cefaclor	100%	100%	100%	100%
Ceftriaxone	33.3%	100%	100%	0%
Imipenem	66.7%	0%	0%	0%
Meropenem	66.7%	100%	0%	100%
Ciprofloxacin	66.7%	100%	0%	0%
Norfloxacin	33.3%	100%	0%	0%
Tobramycins	0%	50%	0%	0%

**Table 3:** Correlation between results of phenotypic tests (disc diffusion, E-test and mCIM) for imipenem.

	Disc Diffusion		Kappa Agreement	*p*-Value
	Susceptible (*n* = 11)	Resistance (*n* = 46)		
E-test				
Susceptible (*n* = 21)	10 (17.5%)	11 (19.3%)	0.498	<0.001 ^
Resistance (*n* = 36)	1 (1.8%)	35 (61.4%)		
mCIM				
Susceptible (*n* = 14)	7 (12.3%)	7 (12.3%)	0.439	0.001 ^
Resistance (*n* = 43)	4 (7.0%)	39 (68.4%)		

^ significant *p*-value.

**Table 4:** Genetic profile of isolated strains.

	*Klebsiella*	*Escherichia*	*Proteus*	*Pseudomonas*	*Acinetobacter*	All Strains
NDM	30 (90.9%)	5 (100%)	3 (75%)	10 (90.9%)	3 (75%)	51 (89.5%)
VIM	2 (6.1%)	0	0	6 (54.5%)	0	8 (14%)
SPM	8 (24.2%)	0	0	2 (18.2%)	0	10 (17.5%)
IMP	3 (9.1%)	1 (20%)	0	0	2 (50%)	6 (10.5%)
GIM	2 (6.1%)	0	0	0	0	2 (3.5%)

**Table 5:** Association between resistance genes and imipenem resistant strains (regarding mCIM).

	*Klebsiella*	*Escherichia*	*Protus*	*Pseudomonas*	*Acinetobacter*
NDM	26 (92.9%)	3 (100%)	2 (100%)	7 (100%)	2 (66.7%)
VIM	2 (7.1%)	0	0	6 (85.7%)	0
SPM	7 (25.0%)	0	0	2 (28.6%)	0
IMP	3 (10.7%)	1 (33.3%)	0	0	2 (66.7%)
GIM	2 (7.1%)	0	0	0	0

**Table 6:** Association between resistance genes and meropenem resistant strains (regarding mCIM).

	*Klebsiella*	*Escherichia*	*Proteus*	*Pseudomonas*	*Acinetobacter*
NDM	20 (90.9%)	2 (100%)	2 (66.7%)	7 (100%)	2 (100%)
VIM	2 (9.1%))	0	0	6 (85.7%)	0
SPM	7 (31.8%)	0	0	2 (28.6%)	0
IMP	2 (9.1%)	0	0	0	1 (50%)
GIM	1 (4.5%)	0	0	0	0

**Table 7:** Agreement between genotypic and phenotypic tests.

	Genotypic	PCR Genes		Kappa Agreement	*p*-Value
Phenotypic		Negative (Susceptible) (*n* = 5)	Positive (Resistant) (*n* = 52)		
E-test					
Susceptible (*n* = 21)		4 (7%)	17 (29.8%)	0.193	0.036 ^
Resistance (*n* = 36)		1 (1.8%)	35 (61.4%)		
mCIM (Meropenem)					
Susceptible (*n* = 21)		2 (3.5%)	19 (33.3%)	0.004	0.878
Resistance (*n* = 36)		3 (5.3%)	33 (57.9%)		
mCIM (Imipenem)					
Susceptible (*n* = 14)		3 (5.3%)	11 (19.3%)	0.040	0.054
Resistance (*n* = 43)		2 (3.5%)	41 (71.9%)		
Disc diffusion (Meropenem)					
Susceptible (*n* = 3)		2 (3.5%)	1 (1.8%)	0.465	<0.001 ^
Resistance (*n* = 54)		3 (5.3%)	51 (89.5%)		
Disc diffusion (Imipenem)					
Susceptible (*n* = 11)		4 (7%)	7 (12.3%)	0.431	<0.001 ^
Resistance (*n* = 46)		1 (1.8%)	45 (78.9%)		

^ significant *p*-value.

**Table 8:** Sequence and expected product sizes of primers used for amplification of *bla*_NDM,VIM,SPM,IMP,GIM_ genes.

Amplicon Size (bp).	Nucleotide Sequence (5’-3’)	Primers
	F (-CGAAAGTCAGGCTGTGTTGCGC-)	
200	R (-GACCGCCCAGATCCTCAACTG-)	*bla*_NDM_
	F (-TCT ACA TGA CCG CGT CTG TC-)	
747	R (-TGT GCT TTG ACA ACG TTC GC-)	*bla*_VIM_
	F (-CTGGCAGGGATCGCTCACTC -)	
604	R (-GGTTTCCGATCAGCCACCTCTCA-)	*bla*_SPM_
	F (-TGAGCAAGTTATCTGTATTC-)	
740	R (-TTAGTTGCTTGGTTTTGATG-)	*bla*_IMP_
	F (-TCCAGAACCTTGACCGAACG-)	
1062	R (-GCCACTCATAGAGCATCGCA-)	*bla*_GIM_

## Data Availability

Not applicable.
